# “The Doctor Helped Me Decide”: A Linguistic Analysis of Contraceptive Shared Decision-Making Narratives in Clinical Encounters Supported by a Decision Aid Mobile Application

**DOI:** 10.5334/pme.2372

**Published:** 2026-02-23

**Authors:** Catherine T. Witkop, Lauren A. Maggio, Abigail Konopasky

**Affiliations:** 1 Departments of Gynecologic Surgery & Obstetrics and Preventive Medicine, Uniformed Services University of the Health Sciences, Bethesda, MD, USA; 2 Department of Medical Education, University of Illinois Chicago, Chicago, IL, USA; 3 Department of Psychiatry, Northwell Health, Glen Oaks, NY, USA

## Abstract

**Introduction:**

Shared decision-making, in which a patient’s values and preferences facilitate the co-construction of a clinical decision, is a key component of patient-centered care and is of pedagogical interest in medical training. Yet, barriers exist to implementation, with limited information about how patients understand and exert agency. To help educators and health professionals facilitate shared decision-making, we examined how patients narrate their agentic experiences and the roles of their physician and a decision aid mobile application (app) in contributing to agency when engaged in contraceptive clinical encounters.

**Methods:**

We conducted a qualitative study of 21 female patients, aged 17–45, who utilized a decision aid app before an encounter for contraceptive services. We conducted linguistic analysis of semi-structured interviews where patients narrated their decision-making experiences, by coding for linguistic markers of agency.

**Results:**

Patients narrated *individual* agency, identifying agentive tasks required for decision-making, including gaining knowledge, asking questions, voicing needs, and making choices. Patients narrated the *app* as a source of information, identifying questions, validating previous knowledge/opinions, and as surrogate provider. Patients narrated *physicians* as supportive agents, cueing patient agency and actions during the visit. *Joint* agency of patient and physician and agency *distributed* across the patient, physician, app, and other resources were contributory.

**Discussion:**

Linguistic analysis can offer important perspectives on patient experiences. The patient, physician, and decision aid app each played roles as agents during shared decision-making. Attending to various roles of agency in clinical encounters and in educational interventions may help health professionals more effectively conduct shared decision-making.

## Introduction

Shared decision-making is a critical component of patient-centered care, with a growing number of practice guidelines calling for its integration into clinical care [1,2] and a required communication skills competency for trainees [3,4]. Despite this, implementation barriers exist, including lack of training and systems-level issues [5], which may be related to the mixed evidence to guide effective teaching strategies for facilitating this complex activity [6]. Further, we propose clinicians often do not understand the patient’s social and behavioral contexts nor the work a patient may have invested prior to the clinical encounter.

Gaps between patient and health professional may be magnified during contraception or other women’s health discussions, where women have reported lack of desired information and inadequate communication regarding options [7] and where gender roles may constrain a woman’s ability to assert agency [8]. While many definitions for agency exist, in this work we are interested in patients’ *sense of agency*: experiences of causation (acting upon others) and autonomy (freedom from constraints). While not always precisely reflective of material circumstances, individuals’ perceptions of their own capabilities and actions feed into motivation and self-efficacy, which are key to patient outcomes [9]. In this study, we examine how participants narrate their encounter with the physician to better understand their sense of agency.

Asymmetries of epistemic access in healthcare interactions play a role in limiting patient-centered care [10]. These asymmetries can be perpetuated through health professions education, as learners are socialized through curricula, assessment, and role modeling that historically privilege certain forms of knowledge over others [11]. Traditionally, the physician has acted as the primary agent in a medical encounter [12], asking pointed questions, performing an examination, making a diagnosis, and prescribing medications or recommending treatment. Although many women plan, prepare, and strategize for clinical encounters, physicians sometimes ignore or undervalue these efforts [13], threatening their sense of agency and future agentic behaviors. Shared decision-making incorporates the patient’s lived experiences and their personal values and preferences, thus aligning with an epistemic justice approach and giving patients a greater sense of agency. Because shared decision-making involves communication between two or more individuals, it is helpful to understand how women experience the complex practice of agency in clinical encounters, investigating their sense of *individual, joint* (two or more agents working together), and *distributed* (two or more agents working in parallel) agency [14,15]. Further, mobile health technology may have a role in distributed agency [16] and mobile apps in particular have been shown to improve health outcomes [17].

Narrative can provide a powerful indicator of patients’ sense of agency. Not just *what* a patient says, but *how* a patient tells their story can provide clues to their experiences of agency [14,18,19]. In particular, a *linguistic* lens on narrative (choices of words, phrases, and structures) [20] can help physicians understand how patients experience causation and autonomy in an encounter, helping them hear the *kinds* of agency patients may experience. This lens brings into focus the complexity of patient agency, not just considering physical action like *using* contraceptives, but *saying* things, *having* certain resources they bring to bear, *being* a certain type of person, or *experiencing* the world in particular ways [19]. This, then, can help health professionals to authentically support *patient* notions of shared decision-making rather than their own. Additionally, from an educational standpoint, such a lens can inform communication skills training, reflective practice, and faculty development by helping clinicians attend to the relational, interdependent elements of agency that are critical for collaboration in healthcare [21].

In our study, we examined contraceptive decision-making, in which attending to a woman’s sense of agency is critically important. This is particularly relevant now, when options related to reproductive health decisions have been curtailed and/or threatened, potentially negatively impacting perceived–or real–agency. While such limitation of reproductive autonomy was most evident in the 2022 Dobbs vs. Women’s Health Organization Supreme Court decision, myriad recent examples can be found in the United States (US) and globally [22]. Previous studies of contraceptive care have identified numerous obstacles and some potential solutions to effective shared decision-making [23,24]. Effective communication skills are key to helping clinicians better understand what supports patients’ sense of agency and successful shared decision-making. Thus, we aimed to understand how a patient’s narrative about their experience in a clinical encounter, preceded by the use of a contraceptive decision aid mobile app, could provide clues for effective shared decision-making and insight into how clinicians can be better educated to recognize and support both patients’ individual agency and the joint exercise of patient-physician agency in this process [21].

## Methods

We conducted a qualitative study in which we interviewed participants and utilized linguistics to analyze how patients narrate their own agency and that of a decision aid mobile app and others (e.g., doctors, family, friends). We focused analytically on their experiences of causation, which imply autonomy (or not) depending upon whether patients narrate themselves or others as causers.

We recruited patients at a walk-in contraceptive clinic at a military medical center in the US between April and July 2019. (See Witkop et al., 2022, for prior published work from this dataset) [25]. Participants included women in the US military who were seen at the clinic and interested in contraceptive services. We excluded participants if they did not speak English, were infertile, currently pregnant, or desired to be pregnant within six months; all other active duty servicewomen between 17 and 45 years of age were eligible and were recruited while they were waiting to be seen for a contraceptive clinic. Twenty-one women consented and participated in the study. After consent, participants downloaded and completed a free contraceptive decision aid mobile app, Decide + Be Ready, on their personal device [26]. This app was developed to enhance epistemic access of medical information to patients and to help patients clarify values and preferences so they can more fully engage as joint decision makers with the health professional. See Witkop et al [26] for detailed description of the app.

While data from the encounter and physician interviews were not included in this current study, for context, physician participants were military and civilian physicians recruited during meetings with clinic staff. The eight physician participants received basic information about navigating the app and had the opportunity to engage with it on their own device but did not receive formal education or training from the study team in shared decision-making.

Immediately following the medical encounter, an associate investigator conducted and audio-recorded a semi-structured interview with the patient. The interview guide was designed to explore the patient’s experience of the preceding visit (Appendix A). Questions elicited perceptions about empowerment to participate in decision-making, how the app and other factors impacted the decision-making process, and past experiences with similar types of health-care decisions. The interview guide was pilot-tested and revised based on feedback and was iterated over the study period based on analysis of participants’ responses. We transcribed and de-identified interviews, which lasted between 12 and 30 minutes.

Using linguistic analysis to examine how patients narrate their interaction with the physician, we coded in Dedoose [27] and then modeled in Excel [28,29]. Based on prior work [30], we coded utterances for linguistic markers of a sense of agency, focusing on who and what patients framed as *grammatical subjects* and, thus, who or what they narrated as causal agents. To better understand how patients narrated the different agents of shared decision-making, we grouped these subjects into categories, based on their role (Table 1).

**Table 1:** Linguistic Categories Used to Characterize Agency in Contraceptive Decision-Making Clinical Encounters.


LINGUISTIC CATEGORY	POTENTIAL SUBJECTS	TYPE OF AGENCY	EXAMPLE

**First-person singular “I” representing the patient**	I	Patient as agent	I decided to get an IUD

**Pronouns and nouns representing the app**	it, the app	App as agent	The app suggested the IUD

**Pronouns and nouns representing the doctor**	she, he, the doctor	Physician as agent	The physician recommended the IUD

**First-person plural “we” representing patient and others (either physician or other women)**	We	Joint agency	We decided on the IUD


Then, following Konopasky & Sheridan [30], we explored reported *causal relationships* between the *I* and *we* subjects and other agents. For example, the patient might say something like, “**The doctor** helped **me** understand,” noting that the doctor caused a change in her understanding. In this instance, agency is *distributed*, “divided up and shared out among multiple people in relation to a single course of action” [31, p.9]. In this way, the patient frames herself as part of a causal chain of agency, committing agentive acts herself but depending upon others (e.g., the app, physicians, family) to also exercise agency.

We recognize that our backgrounds and experiences influenced this study. CW is an obstetrician/gynecologist, who served for over 20 years as a military physician. Her clinical experience and role guided the aim of this study and informed our focus on patient agency and shared decision-making. LM has significant experience in qualitative analysis and AK’s prior work on agency and critical feminist theory provides a unique critical agency perspective on the transcripts. Additionally, as a linguist, AK’s expertise informed our selection and application of linguistic analysis. CW, LM, and AK all bring the additional perspectives of being recipients of women’s healthcare, inside and outside the military setting and hold doctoral degrees in education, which enabled us to consider our findings in the context of health professions education.

Representative quotes were identified to demonstrate particular thematic elements and are reported with each patient participant case represented with a letter in parentheses (e.g., a quote from participant R is denoted as “(R)”). Of note, the term “provider” was used in the original questionnaire, so we use it throughout to refer to a health professional, clinician, or physician; we do not intend it as dismissive of the complexity of the care clinicians provide.

## Results

In response to the question, “Do you feel that you and your provider made a shared decision about your birth control choice?” all but a single patient responded to some degree in the affirmative, endorsing shared decision-making. A sample quote from a patient who felt there was a shared decision was: “Yes… I felt like it was good because I knew sort of what my concerns were specifically, and she addressed each of them and we came up with a plan together.” (R) The single patient who replied in the negative stated that she was clear on her decision prior to the visit: “I don’t think so… this appointment mostly felt like… I want to get the IUD removed and I want to get on the pill and that was pretty much [it]…” (L) One may argue that, even in this scenario, by listening to and agreeing with the patient’s preferences, the physician did engage in shared decision-making.

**Figure 1:**
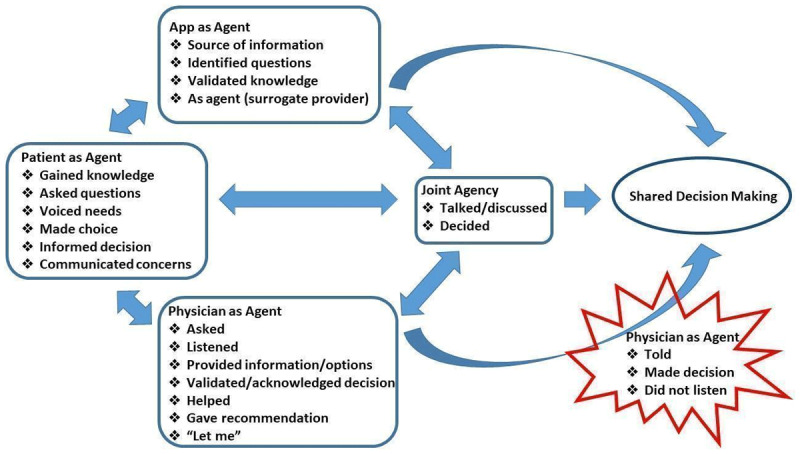
A visual representation of subjects represented in interviews with patients following use of a contraceptive decision aid mobile app and an encounter for contraceptive decision-making. Arrows represent connections between subjects that may facilitate shared decisions and the starburst symbol represents physician actions that may hinder shared decisions.

We describe patterns related to the various subjects represented in the transcripts, including the patient, the app, the physician, and joint agency, which generally encompassed the patient and physician, but sometimes had other referents (e.g., patient and support system). Note that we italicize the type of agency and bold the agent and action to facilitate following the analysis. Figure 1 visually represents our findings.

### A Sense of Individual Agency: Gaining Knowledge and Communicating

In our linguistic analysis of “I” subjects, we learned that women narrated their sense of *individual agency* in unique ways. The specific agentive tasks narrated during the interview varied between patients, but included gaining knowledge, asking questions, voicing needs, making choices, informing the physician of a decision, and communicating other issues or concerns. For example, patient N narrated how she gained knowledge and how that impacted her agency: “after using the app and then going in there and talking with him, **I knew** more about the different types of IUDs and the implant and stuff and so **I knew** going in there what I wanted to talk about, based on what I read in the app.” (N) Patient C focused on inquiry: “So **I asked** him more questions about it. So, once I read the app, **I had questions, I had** more **questions** about it. And then the provider helped me understand more.” (C)

Some women focused on self-expression: “I feel like I played a very important role because **I got to voice my needs** and what I was looking for versus feeling like I didn’t have much of an option.” (B) Two additional agentive tasks were making choices, “he kind of explained and it kind of helped **me make a more informed decision**” (F) and informing the physician: “**I** just **came out** and **said** I want the pill and she’s like okay.” (G) Finally, we identified examples of patients communicating preferences, issues, or concerns. “[the] app was kind of helpful to kind of rack and stack what my priorities were and **to be able to clearly articulate** those to the doctor.” (K) One patient explicitly compares her individual agency to the physician’s, noting that her preferences and voice are driving the decision: “Because they can’t do anything until **I say yes or no**, so the fact that I was like, **I will agree** with you because it is right and it makes sense…he wouldn’t have been able to go any further if **I said no** anyway.” (S)

### A Sense of the App as Agent: Source of Information and Support and Surrogate Provider

By design, the app, which was completed as pre-work before the encounter, was a focal point of all interviews and overall positively described. However, how the patient narrated the role of *the app as an agent* varied and generally fell into four categories. The first was as a source of information: “**the app** actually **stated**…” (A) The second was in identifying questions that the patient can ask the provider: “And **it gave** me the questions I needed to ask my provider. That helps me get on the right path.” (A) The app also helped validate women’s previous knowledge/opinions: “**It** kind of **revalidated**, kind of **helped** me feel better about the one I was thinking about.” (U) Finally, for a few patients, the app was personified as a surrogate provider. For instance, Patient B noted: “**It understands** what you need, and **it gives** you options to choose from, based on your needs. It’s … really simple, **it breaks** everything **down** … it’s fun, it’s animated.” (B)

### A Sense of Physicians as Agents: From Supporting to Hindering Agency

Patients narrated multiple ways that *physicians acted as supportive agents*, cueing or supporting patient agency and actions during the visit. For example, physicians often asked questions: “**She** actually **asked** me quite a few questions…” (E) and the patients noted when physicians listened: “… she was a good sounding board, **she listened**, she was patient with me, she provided me with amplifying information.” (J) The physician often was noted as providing information/options: “And for **him to be open about** … because we do have so many different options and we are at liberty to choose which ones we want.” (S) Sometimes, the physician was noted to validate/acknowledge a decision: “Because when I told her my information and **she agreed** that hey this is the best option for me because…” (O) and “… **she had confidence** that I knew what I wanted.” (L) Women also reported that the physicians helped in various ways: “**She helped** me change my mind.” (G) and “And then the **provider helped** me understand more.” (P) Some patients noted that the physicians gave recommendations, for example: “… I was listened to, the **doctor made recommendations** based on what we discussed…” (K) And, finally, in a few interviews, the patient reported that the physician “let” her do certain things: “She was going to **let me do** Paragard if I really wanted to…” (A) and “It’s probably better that she doesn’t [tell you what to do], that **she lets you choose**, because it’s your body.” (J) While this reflects a sense that the physician still has more power in the relationship (i.e., “letting” signifying the physician is the one with the power to give permission rather than the patient inherently having it), these provider actions were nonetheless narrated as supportive of women’s agency.

Yet, providers’ actions were not always supportive of patient agency. For example, several women reflected on past experiences, often contrasting an example with what they describe as a more agentive experience this time around. Examples of past provider behaviors that they perceived hindered their agency included: “told”, “made decision(s)”, “did not listen.” These are represented in Figure 1 as negatively impacting the contribution of the physician in supporting shared decision-making, as seen in these three examples: “… **they don’t really work with you. They tell you** what they have to offer and then if you don’t want to deal with that, it’s not your loss” (B); “**They made the decision** for me which turned out to be… a bad decision.” (O)

### A Sense of Joint Agency: Patients Together with Providers or their External Support Systems

“We” subjects, when referring to the patient and physician, demonstrated *joint agency*, where the patient narrated a joint action that the patient and physician undertook together. This sense of joint agency was not observed across all patients, even when a woman reported shared decision-making; joint agency emerged in only about half of the cases we observed. These joint “we” examples with providers fell into two general categories: talking/discussing and deciding. Examples of talking/discussing included: “**We did talk** about the options and what was best suited for my situation at the time… the doctor made recommendations based on what **we discussed**.” (K) and “… but **we did discuss** the other options.” (F) In terms of jointly deciding, some examples were: “Using the app, I felt more informed about the methods, and then communicating with the doctor my values and preferences, **we were able to pinpoint** specifically which one would be best for my care and hopefully long-term care.” (M) and “So I felt like it was good because I knew sort of what my concerns were, specifically, and she addressed each of [them] and **we came up with a plan** together.” (R)

We also observed “we” referring to patient joint actions with those *other* than the physician. Even though, in our model, this may not be directly related to shared decision-making, it is an important type of joint agency that may contribute to how women understand their own agency as patients. In these data, “we” often referred to the patient together with other women who work or other military women (those “like me”), indicating a joint experience, not necessarily shared decision-making. In this case, the verbs were often related to deploying, or having/not having something (access, knowledge, ability to participate, control). For example, referring to herself and other women, patient S says, “But if it is something **we have access** to prior, **we can look at it** and **take our time to say** okay, here we go …” (S) “We” was also used to refer to the patient together with family members or partners, for example, patient Q stated: “… me and my husband, **we sat and had a discussion**…” (Q)

### A Sense of Distributed Agency: A Complex System

In contrast to individual and joint agency, *distributed agency* narrates multiple agents working separately on actions toward the same goal (in this case, usually deciding on contraception), often with reported causal relationships between them. Following the arrows in Figure 1 demonstrates the numerous ways distributed agency was represented across the interviews. Almost all participants narrated distributed agency during their interviews, but which agents were involved varied. In several cases, women (“I” as subject) narrated distributed agency with the app alone. For example, patient C said the app “gave” information and questions for *her* to ask and described it as a source of low-stakes support to her which helped in exercising agency with the physician. She reports that in the past, she “felt pressure” during medical encounters, but did not in this case. (C) In other cases, the patient reported distributed agency with the physician, which sometimes ended with joint agency: “Because when **I told** her my information and **she agreed** that hey this is the best option for me because it’s the reduction in the hormones and **she also gave me** other options that could be a possibility, so **we went** with what **I wanted**.” (O) The back-and-forth of the separate actions of the patient and physician here culminate in the joint agency of a decision based on patient desires.

In some cases, agency was distributed across the patient, physician, and app. Women narrated scenarios in which each of the three had roles that depended somewhat on the actions/impact of the others. For example, Patient P, referencing the app, stated “**it was telling** me about the IUDs.” (P) Then, the patient stated: “Once **he** [provider] **asked…** By the time **I read the app, I** had already **changed my mind**… **I thought** about changing my mind to the other one and once **he asked** those questions… Once **I asked** those questions to him, **he helped** and then **I changed** and then at the last minute **I decided** I wanted to get the hormonal…after **I looked at the app**.” (P) This patient explained the decision-making process as a sequential series of actions by the physician, the app, and herself. The app helped the patient understand her options and come up with questions. The physician responded to the questions and the resulting conversation evolved into shared decision-making.

There were also examples of the app *starting* the chain of agency (follow arrows in Figure 1). For patient J, the app provided information and questions to ask; the patient then had a conversation with the physician who subsequently “let” the patient make the decision: “I was concerned about mood and **the app confirmed** that the IUD has no traceable side effects to mood or depression or anything like that, so in my meeting **I was able to say**, hey, I know this to be true, having learned about the IUD and talked about it with my provider before and I just saw it on the app…. Well when **I asked** questions about the individual methods, or my decision today which was to keep the IUD or get it removed, **she heard** me out on reasons why I was waffling on that and then [**she**] **provided me** with more medical back up for why it’s safe and side effects and things like that and then [**she**] **let me** make the choice.” (J)

## Discussion

Our study surfaced several patterns in patients’ sense of agency. The concept of agency is critical to understanding the complexity of shared decision-making and women see contraception as an important outlet for exerting agency. Yet, because agency differs across individuals, we must understand how women narrate perceptions of their own agency and the influence of others (including tools such as the app) on that perceived agency. Health professionals who are intentional about eliciting and understanding their patients’ social and behavioral contexts as well as their patients’ prior work, may be more effective in conducting shared decision-making encounters. Therefore, in this discussion, we focus on how recommendations arising from our findings can be intentionally integrated into health professions education to help clinicians more successfully facilitate shared decision-making with women. (See Table 2 for a summary of recommendations).

**Table 2:** The Linguistic Lens on Agency: Recommendations for Providers.


TYPE OF AGENCY	RECOMMENDATION	TARGET LANGUAGE

**Patient individual agency**	Ask open-ended questions and give patient time to express questions, needs, concerns, thoughts	***Patient:*** - “I know/don’t know…” - “What/how/why/do…?” - “I need/want…” - “I think…”

**External resource agency (e.g., apps, websites)**	Identify and make available decision aids (including apps or other resources) that facilitate shared decision making	***Provider:*** - “What did you learn from the [resource] about your preferences? What questions do you have for me?” ***Patient:*** - “It [resource] stated/suggested…” (followed by question for provider)

**Physician agency (supportive)**	Ask questions to determine patient knowledge and needs; validate patient decisions; listen actively.	***Provider:*** - “What/how/why/do *you*…?” - “What is most important to *you* in this decision?” - “I agree with *your* decision to…” - “Tell me more…”

**Physician agency (hindering)**	Do not use directives or make decisions for the patient	***Provider—to AVOID:*** - “You’re/We’re going to do…” - “I’ve decided that…”

**Joint patient-provider agency**	Listen for patient use of “we” but do not initiate it unless used by patient.	***Patient:*** - “I/we should do…” (***provider*** follows patient lead)

**Distributed agency (across patient, provider and/or other resources)**	Consider “three-talk” model to enhance autonomy. Cue and listen for patient values, preferences, and sociocultural context. Avoid directives.	***Patient-Provider:*** - “Frequent turn-switching between patient and provider - “This resource suggested…” - “My friend/partner and I talked about…”


The patient, the app, and the physician each played a significant role as agents in patient narratives of the encounter. Since women may sometimes view contraception negatively, despite also seeing it as an outlet for exerting agency [8], we explore how we can facilitate women’s roles as *individual* agents in this process. One recommendation is that clinicians should be trained to intentionally ensure that patients have the opportunity to engage in the agentive actions that they narrated for themselves (e.g., gaining knowledge, voicing needs), perhaps even cueing these actions if they do not see patients initiating them. For example, as a system-improvement, resources such as a decision aid can be made available to patients in advance of appointments, e.g., through a patient portal or website. During the visit, the provider should ask about and respond to women’s agentive pre-appointment work, both for decision-making and ultimately for reproductive justice [13,32]. Incorporating training to encourage this intentionality will help to normalize it within physician practice.

A second recommendation centers on recognizing and supporting *distributed* and joint agency, particularly when non-human agents such as decision aids are involved. In our study, as a specific example of a resource as agent, the app played a key role in supporting these women’s sense of agency, consistent with the concept that non-human agents have capacity to produce an effect, studied extensively for interaction design [33]. Our participants perceived the decision aid as *helpful* and, in some cases, serving *itself as an agent* in the decision-making process. Engagement with the app also provided many of the patients with questions to ask the physician during the encounter--and that act of inquiry led to distributed agency in almost all encounters and to joint agency in multiple cases. Although we cannot extrapolate our findings to all decision aids, those containing certain standard elements (e.g., the provision of knowledge using pictorial representations of risks/benefits and the patient’s active engagement in identifying values and preferences) may be most effective at facilitating shared decision-making [34,35]. From an educational standpoint, these findings highlight the importance of teaching clinicians to integrate patient-facing tools into encounters rather than treating them as standalone information tools. For example, educators can model and encourage language for trainees such as: “What did you learn from the app about your preferences?” or “What questions came up for you as you reviewed the app’s information?” Such language, cuing distributed and joint agency, can be taught, practiced, and reinforced through simulation, observed clinical encounters, and feedback, helping learners recognize decision aids as partners in relational agency rather than as standalone informational tools.

A third set of recommendations involves clinicians’ use of language and their attention to their responsibility in shared decision-making. Providers can actively monitor themselves during encounters to determine if they are performing the supportive agentive actions women narrated (e.g., asking, listening, validating decisions). Additionally, since we learned that shared decision-making does not necessarily require evidence of joint agency (only present in about half of the narratives), providers’ use of “we” may need to be tempered; not all patients will relate or commit to a joint action. Further, the use of “we” can lead to ambiguity in decisional responsibility as Gulbrandsen [36] previously described. Our findings align with those of a previous study, which found that greater use of first-person pronouns during a clinical encounter was associated with patient satisfaction measures [37]. Therefore, rather than encouraging uniform use of “we”, educators can help clinicians develop sensitivity to patient cues, including patient use of “I” and “we” and adapt their language accordingly during the encounter. This reframes patient pronoun use as a teachable communication behavior that can be observed, discussed, and refined through feedback.

Patients’ narration of *distributed agency* could also inform how educators teach existing shared decision-making frameworks, such as the “three-talk” model. This model, which was developed to enhance a patient’s “autonomous capacity,” focuses on the emotional and relational aspects of care and supporting the patient during the decision-making process [5,38]. Moreover, it represents shared decision-making as “a fluid transition” between “team talk,” “option talk” and “decision talk” [5]. While this model provides a high-level conceptual framework for shared decision-making, our distributed agency findings provide educators with concrete insights into to *how* they can encourage learners to engage in each type of “talk” to support patient agency. Using suggested language to facilitate women’s agency will shape that provider’s awareness of the importance of patient values, preferences, and sociocultural context. For instance, providing information or recommendations may spur patients’ agentive actions like communicating concerns or making a choice; conversely, telling, making the decision, and not listening may hinder such actions. Educators can use patient narratives and suggested language as instructional tools to help learners recognize how their communication choices shape opportunities for agency.

While the “three talk” model provides a framework, communication skills require practice, suggesting that health professions education has a central role in translating these recommendations into routine clinical behaviors that are familiar and increasingly natural. Educational interventions aimed at improving shared decision-making can and should include the use of simulations and standardized patients; feedback from standardized patients; and educators observing simulated encounters. Such approaches allow learners to practice recognizing and supporting agency in a safe, low-stakes environment, where they can receive targeted feedback on their communicative behavior.

Finally, given clinicians’ historical role in reproductive injustice, medical educators must bear responsibility for explicitly teaching towards reproductive justice in contraceptive care [39,40]. Supporting agency is not merely a desirable communication skill, but a core educational competency [3,4] that must be intentionally taught, modelled, and reinforced across training. By centering and supporting agency within shared decision making, education becomes a vital part of the equation in advancing equitable, patient-centered care.

### Limitations

Although we report on how the physician and the app contribute to patient agency and shared decision-making, we do not report on how the patient narrates the community (including her partner, friends, family, other military women) in her decision-making. We offered some brief mentions of joint agency with others, but did not delve, in the interest of space, into additional evidence of others’ roles in clinical decision-making outside the encounter.

## Conclusion

We have used linguistic tools to reveal important insights into women’s experiences with and understandings of contraceptive care and how they interact with healthcare providers, offering recommendations for providers. What providers intend to say to women patients may not always be what patients hear. By understanding how their words come across, providers may be able to invite women more fully into decision-making. For health professions education, these findings highlight the importance of explicitly teaching, practicing, and reflecting on patients’ language use and its connection to individual, distributed, and joint agency as a core component of shared decision-making training.

## Appendix A: Patient Interview Guide

This guide will be used to interview patients following the visit for contraceptive counseling in which the decision aid was provided to the patient to work through and bring to the visit. The provider also had access to the decision aid prior to the visit.

Notes for the research assistant: Introduce yourself and your role in the study to the interviewee. Provide information about yourself so that the interviewee understands how your background may or may not be relevant to the interview.

For each of the following questions, use the numbered questions to guide the interview and use the bulleted questions as probes if needed. Once each of the bulleted points has been addressed (either because interviewee offered it up or you have asked the probe), move on to the next numbered question. If an interviewee answers one of the numbered questions while answering another numbered question, there is no need to repeat the question.

Please record the interview and ensure that voices can be clearly heard on the recording.

**Thank you again for participating in the study. My name is [X], and I am a research associate on the study. This next part of the study will be a 15–20 minute interview. During the interview I will ask you some open-ended questions about the contraceptive application itself, how the app impacted your conversation with your provider, and some questions that are specific to being a servicemember in relation to the study. Just as a reminder we will be recording the interview, and I am going to start recording now…**

### Section 1

**Let’s begin with some questions about the use of the app in your discussion of contraceptive options**.

**1. Approximately how much time in total did you spend using the app before the appointment?**
**2. Your preferences about your birth control method have to do with your personal choice about your method. In what ways, if any, did the app impact your ability to identify and communicate your preferences to your provider?**
  *This question addresses the patient’s perceived ability to identify and communicate preferences*.
**3. Your values have to do with the amount of weight you place on a particular characteristic of a contraceptive method. Did you feel empowered to talk about your own values with your provider?**
  *This question addresses the patient’s perceived ability to identify and communicate preferences*.
  **If yes, in what ways, if any, did the app contribute to you feeling empowered to talk about your preferences or your values?**
  *This question addresses the patient’s comfort in communicating with the provider*.
**4. Do you feel that you and your provider made a shared decision about your birth control choice? Why or why not?**
**5. How comfortable are you with your decision about your birth control choice?**
  *These questions get at the patient’s perception of the shared decision-making process and therefore addresses the perceived effectiveness of the tool in enhancing shared decision making*.
**6. Do you feel that you had an important role in the decision about your birth control method?**
  *This question gets at the patient’s feelings about having agency in the decision-making process and therefore addresses the effectiveness of the tool in enhancing patient agency*.

### Section 2 (check time)

**Thank you, now let’s move onto some questions more specific to being a servicemember in relation to the study**.

**7. First, which Service are you in?**
**8. Second, in which of the following categories for your rank do you fall: E1-E3, E4-E6, O1-O3, O4-O9, Warrant Officer W1-W5**
**9. What, if any, military-relevant concerns or issues influenced your contraceptive choice?**
  - **For example, did you have any concerns related to a contraceptive option that were related to your assigned duties in the military?**
  *This question gets at actually identifying whether or not the patient identified and/or expressed military-relevant patient values*.
**10. In some health situations you may have several options for care. For example, perhaps you could choose one therapy or another therapy. As a service member, how much input do you feel you generally have in making decisions in these types of situations?**
  - **Think about the last time you had to make a medical decision. Did you feel empowered to make the decision?**
  - **Can you think of a health situation when you felt as though you did not have a say in the decision, but felt like you should? Tell me about it**.
  *This question gets at the patient’s feelings about having a say in preference-sensitive decisions*.
**11. This app was meant to help you and your provider with shared decision making around a medical decision. Would you like to use an app like this (or another such tool) in the future for other medical decisions? Why or why not?** *If yes***, For what types of decisions do you think it would be most valuable to you?**
  *This open-ended question provides the interviewee an opportunity to share any additional thoughts about their interest in participating in shared decision making in the future*.
